# Moderate‐intensity exercise increases renalase levels in the blood and skeletal muscle of rats

**DOI:** 10.1002/2211-5463.12812

**Published:** 2020-04-21

**Authors:** Katsuyuki Tokinoya, Yasuko Yoshida, Takehito Sugasawa, Kazuhiro Takekoshi

**Affiliations:** ^1^ Graduate School of Comprehensive Human Sciences University of Tsukuba Japan; ^2^ Japan Society for the Promotion of Science Tokyo Japan; ^3^ Department of Medical Technology Faculty of Health Sciences Tsukuba International University Japan; ^4^ Division of Clinical Medicine Faculty of Medicine University of Tsukuba Japan

**Keywords:** catecholamine, exercise, kidney, renalase, Sp1, STAT3

## Abstract

Renalase is predominantly expressed in the kidney, where it plays a role in catecholamine metabolism and blood pressure regulation. Moderate‐intensity exercise (MEX) has been shown to increase the concentration of renalase in the blood and to reduce renal function in humans. Moreover, such exercise was also reported to increase catecholamine levels. Here, we examined renalase concentration in the blood and renalase expression levels in different organs after MEX in rats. Twelve male Wistar rats were made to run on a treadmill (MEX group) for 60 min at 20 m·min^−1^, after resting for 15 min. The control group rats were euthanized after resting on the treadmill. Tissue and blood samples were analyzed using western blotting, real‐time RT‐PCR and ELISA. Overall, the concentrations of renalase in the blood were significantly higher in the MEX group than that in the control group. Renalase expression was decreased in the kidney after 60 min of exercise, whereas the expression of renalase mRNA and protein in the extensor digitorum longus and plantaris muscles, respectively, increased after exercise. However, the expression of renalase in the other tissues examined did not change after acute exercise. In conclusion, we report that MEX for 60 min increases both renalase concentration in the blood and its expression in skeletal muscle.

## Graphical abstract

The concentration of renalase in the blood and its expression in skeletal muscle were significantly increased in rats that underwent moderate‐intensity exercise as compared with the sedentary group, whereas renalase expression in the kidney decreased after exercise. Renalase expression in other tissues did not change after acute exercise.

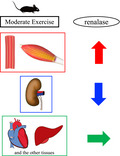

**Abbreviations**
ANOVA: analysis of variance
CON: control
*C*_t_: cycle threshold
EDL: extensor digitorum longus
GAPDH: glyceraldehyde‐3‐phosphate dehydrogenase
MEX: moderate‐intensity exercise
SD: standard deviation
Sp1: specificity protein 1
STAT3: signal transducer and activator of transcription 3
ZBP89: zinc binding protein 89

Renalase is a recently discovered FAD‐dependent soluble monoamine oxidase [1]. Its primary functions include catecholamine metabolism and blood pressure regulation [1, 2, 3]. Although renalase is predominantly expressed in the proximal tubule in the kidney, its expression has also been observed in other tissues, including the skeletal muscle, heart and intestine [1]. In addition to its function in catecholamine metabolism, renalase was recently reported to inhibit apoptosis, inflammation and fibrosis [4, 5, 6].

Renalase expression is increased by epinephrine in human renal proximal tubular epithelial (HK‐2) cells and C2C12 myotubes [7, 8]. It has been reported that three transcription factors, that is, specificity protein 1 (Sp1), signal transducer and activator of transcription 3 (STAT3) and zinc binding protein 89 (ZBP89), regulate renalase expression. In addition, the expression of these factors has been shown to be increased in response to epinephrine treatment in HEK293 cells [9]. Moreover, several other transcription factors have been shown to be related to renalase expression. The concentration of renalase is correlated to that of catecholamine in the blood of patients with chronic kidney disease [10].

Exercise affects the expression of renalase [11, 12, 13]. Our previous reports showed that the serum renalase concentration significantly increased after long‐distance running at a moderate velocity in humans [12]. In addition, a negative correlation was observed between the concentration of renalase in the blood and renal function. This indicates that renal function deteriorates with acute exercise. Tokinoya *et al*. [11] showed that the expression of renalase protein increased in the skeletal muscle and decreased in the kidney after acute exercise for 30 min; however, the blood concentration of renalase did not change under the same conditions. They discussed that the increased expression of renalase in the skeletal muscle may be offset by decreased expression in the kidney. Further, Czarkowska‐Paczek *et al*. [13] reported that the mRNA content of renalase decreased in the white portion of the gastrocnemius muscle of rats after running on a treadmill for 60 min at 28 m·min^−1^. This speed was defined as ‘high intensity’ by Soya *et al*. [14], during which the white skeletal muscle fibers are primarily active [15]. However, these previous studies did not examine renalase expression in the context of moderate‐intensity exercise (MEX). Plasma catecholamine levels have been shown to increase depending on exercise intensity [16]. In addition, epinephrine and norepinephrine concentrations have been reported to be significantly increased after MEX (60% of maximal oxygen uptake) for 60 min [17]. Thus, MEX increases catecholamine concentration.

In this study, we aimed to measure renalase concentration in the blood and its expression in the skeletal muscle after MEX for 60 min.

## Materials and methods

### Ethics statement and experimental design

The Animal Ethics Committee of The University of Tsukuba approved all experimental protocols, which were performed in accordance with the principles and guidelines on animal care proposed by the Physiological Society of Japan (approval number: 17‐076).

Eight‐week‐old male Wistar rats (Japan SLC, Inc., Shizuoka, Japan) were used for the experiments (weight, 166–187 g). The rats were housed in a room at 20–26 °C and 40–60% humidity, with a 12 / 12‐h light/dark cycle. Animals were fed a normal chow diet (MF 12 mm φ pellet; Oriental Yeast Co., Tokyo, Japan) and were given *ad libitum* access to water. These rats were housed for 1 week to acclimate them to their new environment.

**Fig. 1:**
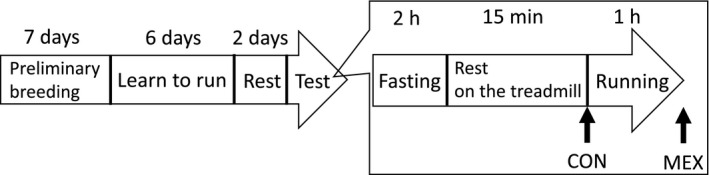
Experimental design. *n* = 6 per group. The arrow indicates the sampling point in each group.

The experimental design is shown in Fig. 1. All rats were familiarized with the exercise regimen on a motor‐driven horizontal treadmill (FVRO.4E9S‐6; Fuji Medical Science Co. Ltd., Chiba, Japan) for 30 min using mild electric shocks (0.8 mA) at the rear end of the treadmill and were initiated on a 6‐day program. After the final exercise session, rats were allowed 48 h of rest and then were divided into the following two groups: control (CON; *n* = 6) and MEX (*n* = 6). The weight of the rats in the two groups did not differ significantly (CON, 252.2 ± 4.3 g; MEX, 247.8 ± 6.5 g; *P* = 0.59). These rats were fasted for 2 h and then allowed to rest on a treadmill for 15 min. Subsequently, the CON group rats were euthanized under isoflurane‐induced anesthesia. Rats in the MEX group ran for 1 h at a moderate intensity and were then euthanized under anesthesia immediately after running. An acute bout of exercise comprised treadmill running for 1 h at a speed of 20 m·min^−1^. This speed was referred to as ‘moderate‐intensity running’ by Soya *et al*. [14]. Tissue samples were collected from the muscle, kidney, heart, liver and lung. Plasma was collected and treated with EDTA. Samples were stored at −80 °C for subsequent analyses.

### Western blotting

Radioimmunoprecipitation assay buffer (1 m Tris–HCl, 5 m NaCl, 0.5 m EDTA, 0.5% SDS, 2.5% sodium deoxycholate, 5% Nonidet P‐40, and distilled water) was added to the tissue samples, and the mixture was homogenized using a bead crusher (Tissue Lyser; Qiagen, Hilden, Germany). After homogenizing at a frequency of 25 Hz for approximately 2–5 min, the samples were centrifuged (4 °C, 12 000 ***g***, 15 min), and the supernatant was collected. A bicinchoninic acid assay kit (Takara Bio Inc., Kusatsu, Japan) was used to determine the protein concentration, using a standard curve of bovine serum albumin. Homogenized tissue samples were diluted in radioimmunoprecipitation assay buffer (skeletal muscle, 30‐ to 50‐fold dilution; kidney, 50‐fold dilution), and 10 μL of the diluted sample was added to each well of a microplate, along with 100 μL of the working solution containing reagent A and reagent B mixed in a ratio of 50 : 1. Absorbance was measured at 562 nm on a microplate reader (Varioskan LUX; Thermo Fisher Scientific, Waltham, MA, USA) after incubation for 10 min at 37 °C.

Samples were prepared by diluting the supernatant of homogenized tissue samples with sample buffer (2× Laemmli sample buffer and 10% 2‐mercaptoethanol) to a concentration of 2 mg·mL^−1^. Five microliters of each sample was used for SDS/PAGE. After proteins were resolved by electrophoresis, they were transferred to a polyvinylidene fluoride membrane presoaked with methanol (GE Healthcare Life Sciences, Chicago, IL, USA). Polyvinylidene fluoride membranes were blocked for 30 or 180 min with 5% skim milk in TBS‐T (0.1% Tween 20) containing 5% Blocking One (Nacalai Tesque, Kyoto, Japan). Different blocking times were used for the membranes because the primary antibodies differed in their specificities. A blocking time of 180 min for the anti‐renalase serum has been reported in a previous study [18], and a blocking time of 30 min was used for anti‐glyceraldehyde‐3‐phosphate dehydrogenase (GAPDH) serum to avoid detection of nonspecific bands. Membranes were incubated overnight with the primary antibodies against renalase (ab178700; Abcam, Cambridge, UK) and GAPDH (internal CON; sc‐365062; Santa Cruz Biotechnology, Dallas, TX, USA) at 4 °C with continuous shaking. They were then washed three times in TBS‐T for 5 min each and incubated with horseradish peroxidase‐conjugated secondary antibodies for 60 min at 25 °C. The membranes were then washed three more times in TBS‐T for 5 min each, incubated with the chemiluminescent reagent (NEL103001EA; PerkinElmer, Waltham, MA, USA) and imaged using an ImageQuant LAS‐4000 system (GE Healthcare Life Sciences). Signal intensities were analyzed using justtlc software (Sweday, Sodra Sandby, Sweden). All primary and secondary antibodies were diluted 1 : 1000 and 1 : 10 000 with TBS‐T containing 5% Blocking One. The signals for bands corresponding to those of renalase and GAPDH (internal CON) were measured and analyzed.

### ELISA

Plasma renalase concentration was measured using an FAD‐dependent amine oxidase ELISA kit (Cloud‐Clone Corp., Houston, TX, USA). All assays were performed according to the manufacturer’s instructions. Plasma samples were diluted 100‐fold with PBS immediately before ELISA analysis. Absorbance was measured at 450 nm on a microplate reader (Varioskan LUX). Renalase concentration in the plasma was quantified by comparing absorbance values with those on a calibration curve prepared using a dilution series of the standard. However, we could not determine renalase concentration in one of the rats from the MEX group.

### Quantitative real‐time RT‐PCR

mRNA was isolated from skeletal muscle, kidney, heart, liver and lung tissues. The tissue samples were first homogenized in a TissueLyser bead mixer (Qiagen) at a frequency of 25 Hz for 2–5 min. Total mRNA was isolated using the Sepasol‐RNAⅠSuper G kit (Nacalai Tesque), according to the manufacturer’s instructions. Total RNA concentration was determined by measuring absorbance at 260 nm using an ND‐1000 spectrophotometer (NanoDrop; Thermo Fisher Scientific). mRNA samples were stored at −80°C for further analyses.

Total RNA was reverse transcribed into cDNA using PrimeScript RT master mix (Perfect Real Time; Takara Bio Inc.). To quantify gene expression levels, we performed PCR using a KAPA SYBR FAST qPCR kit (Kapa Biosystems, Wilmington, MA, USA) on an Applied Biosystems 7500/7500 Fast Real‐Time PCR System (Thermo Fisher Scientific), according to the manufacturer’s instructions.

The cycling program included an initial denaturation at 95 °C for 20 s, followed by 40 cycles of denaturation at 95 °C for 3 s, and annealing and elongation at 60 °C for 3 s. A melting curve analysis confirmed that the PCR product did not contain nonspecific by‐products. *GAPDH* was used as an internal CON for normalizing the mRNA contents. The mRNA content of renalase in each tissue was determined using the standard curve method. The cycle threshold (*C*_t_) value of the target gene mRNAs was normalized to the *C*_t_ value of *GAPDH* mRNA (ΔΔ*C*_t_ method) for both the CON and MEX groups. The primer sequences used in this study are shown in Table 1.

**Table 1:** Primer sequences used for mRNA analyses. *Zfp148*, zinc finger protein 148.

Name	Forward (5′→3′)	Reverse (5′→3′)
*Gapdh*	GGAAACCCATCACCATCTTC	GTGGTTCACACCCATCACAA
*Renalase*	TGCCAACAGTCCTCATAATCC	TCCTTCCTTCACTTCCATTCC
*Sp1*	GCTATAGCAAACACCCCAGGT	CAGGGCTGTTCTCTCCTTCTT
*Stat3*	CCTTGGATTGAGAGCCAAGAT	ACCAGAGTGGCGTGTGACT
*Zfp148*	CCCTTCCTTGAAGTCTTCGTT	CCAGTTTGTCGTCAATGTTCA

### Statistical analysis

Data are shown as mean ± standard deviation (SD). For all measurements, a one‐way analysis of variance (ANOVA) was used to evaluate significance. Statistical analyses were performed using spss statistical software (version 24.0; SPSS Inc., Chicago, IL, USA). A Student’s *t*‐test was used for comparison between the two groups, with *P* values <0.05 considered significant.

## Results

### Renalase mRNA contents in skeletal muscles

**Fig. 2:**
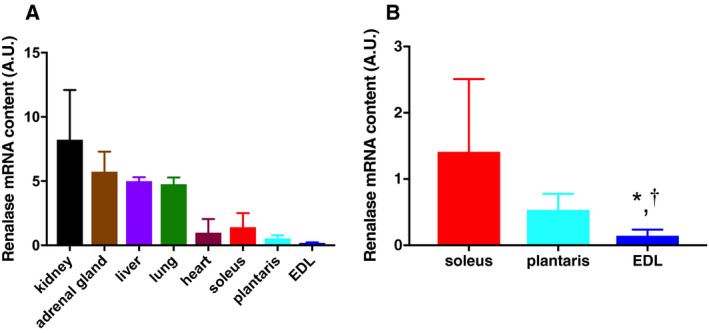
Renalase mRNA contents in skeletal muscles and the other tissues. The mRNA contents of renalase were assessed in skeletal muscles (B) and the other tissues (A) using the standard curve method. Data are shown as mean ± SD. *n* = 6 in the CON group. Data were analyzed using a one‐way ANOVA (only B). *P < 0.05 versus plantaris; ^†^ *P* < 0.1 versus soleus.

The mRNA content levels of renalase in the kidney, heart, liver, lung, adrenal gland and skeletal muscle were examined in the CON group rats using real‐time PCR (Fig. 2A). As shown in Fig. 1, the mRNA content levels of renalase differed in different skeletal muscle fibers (Fig. 2B). Renalase expression levels were approximately 10‐fold higher in the soleus muscle than that in the extensor digitorum longus (EDL) muscle. In addition, renalase expression was higher in the plantaris muscle than that in the EDL muscle.

### Tissue and plasma renalase levels after exercise

**Fig. 3:**
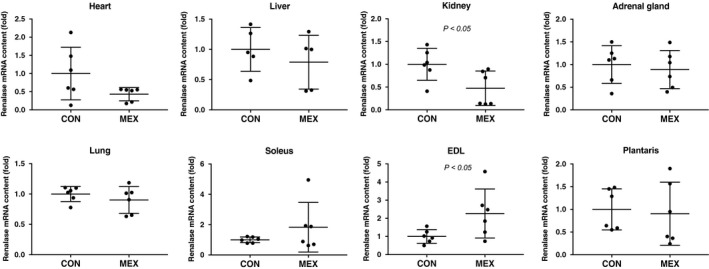
Renalase mRNA contents in each tissue after MEX. The mRNA content of renalase in each tissue was compared between the CON and the MEX groups using the ΔΔ*C*_t_ method. Data are shown as mean ± SD. *n* = 5 or 6 per group. Data were analyzed using a Student’s *t*‐test. Statistical significance was set at *P* < 0.05 between the CON versus MEX groups.

**Fig. 4:**
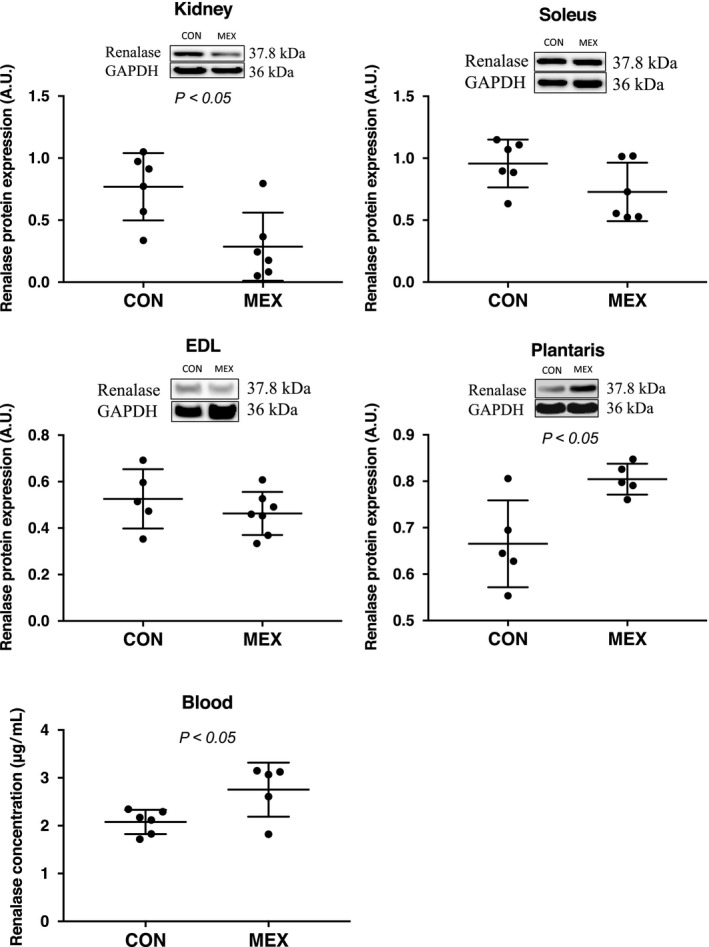
Tissue expression levels and concentration of renalase in the blood after MEX. The expression of renalase, at the protein level, in the skeletal muscles was compared between the CON and MEX groups using western blotting. The concentration of renalase in the blood was measured by ELISA. Data are shown as mean ± SD. *n* = 5 or 6 per group. Data were analyzed using a Student’s *t*‐test. Statistical significance was set at *P* < 0.05 between the CON versus MEX groups.

The effect of MEX on the mRNA content of renalase was investigated using the ΔΔ*C*_t_ method. There were no significant differences in renalase expression in the heart, liver, lung or adrenal glands between the CON and MEX groups (Fig. 3). However, mRNA contents of renalase in the kidney were significantly lower in the MEX group than that in the CON group (*P* < 0.05; Fig. 3). In addition, the expression of the renalase protein in the kidney decreased after exercise (Fig. 4). There was no significant difference in the mRNA content of renalase between the soleus and plantaris muscles. However, the mRNA content of renalase in the EDL muscle was significantly higher in the MEX group than in the CON group (*P* < 0.05; Fig. 3). Furthermore, the expression of renalase protein in the plantaris muscle significantly increased after exercise (Fig. 4). Plasma renalase concentration was significantly higher in the MEX group than that in the CON group (*P* < 0.05; Fig. 4).

### Transcriptional regulation of renalase in the skeletal muscle

**Fig. 5:**
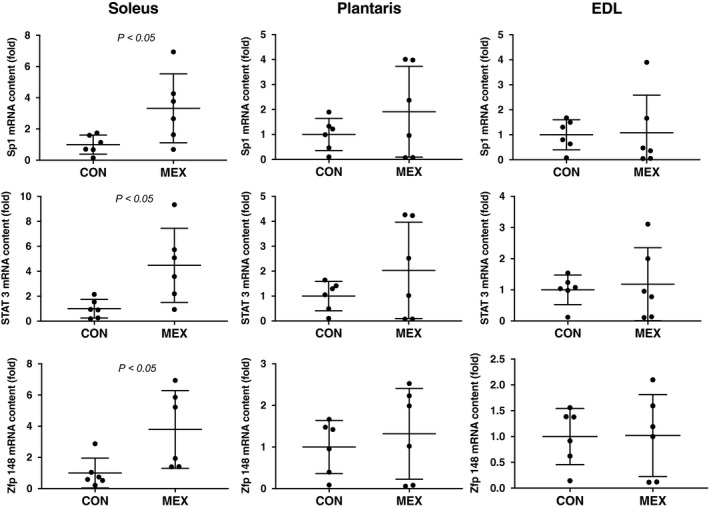
The mRNA contents of *Sp1*, *STAT3* and *ZBP89* in each skeletal muscle fiber type after MEX. The mRNA contents of *Sp1*, *Stat3* and *Zfp148* (*ZBP89*) in skeletal muscles were compared between the CON and MEX groups using the ΔΔ*C*_t_ method. Data are shown as mean ± SD. *n* = 6 per group. Data were analyzed using a Student’s *t*‐test. Statistical difference was set at *P *< 0.05 between the CON versus MEX groups.

The mRNA content of *Sp1*, *ZBP‐89* and *STAT3* in the skeletal, soleus, plantaris and EDL muscles was compared between the CON and MEX groups. The expression of all three mRNA transcripts was significantly higher after exercise only in the soleus muscle (*P* < 0.05 for all transcripts; Fig. 5).

## Discussion

This study aimed to determine the renalase concentration in the blood and renalase expression in different organs after acute exercise at moderate intensity for 60 min. In addition, we measured the expression of transcription factors that might be involved in regulating the expression of renalase in the skeletal muscles. First, renalase was found to be expressed in skeletal muscles, which led us to compare its expression among the different skeletal muscle fiber types. The mRNA content of renalase in the skeletal muscle fiber types is not known, although a previous study has reported that the expression of renalase protein in the skeletal muscle fiber types after acute exercise is dependent on the intensity of the exercise. To the best of our knowledge, this is the first study that shows significant differences in renalase mRNA contents among various muscle fiber types. The soleus muscle (red muscle‐dominant rich in myoglobin and mitochondria) showed approximately 10‐fold higher mRNA content of renalase than the EDL muscle (white muscle rich in glycolytic enzymes; Fig. 2B). In addition, the plantaris muscle showed significantly higher renalase expression levels than the EDL muscle. Therefore, the mRNA content of renalase in skeletal muscle might differ based on the fiber type. However, the mechanism responsible for this difference was unclear in this study.

In this study, the concentration of renalase in the blood was significantly higher in the MEX group than that in the CON group (Fig. 4). Our study design involved longer exercise durations than the previous study [11], and exercise was performed at a moderate intensity to ensure the release of catecholamines. We consequently showed that the levels of renalase in the blood increased significantly, which was similar to observations in human subjects [12]. We also investigated exercise‐induced variability in renalase mRNA contents in the visceral organs and skeletal muscles. No significant differences in renalase mRNA contents in the heart, liver, lung or adrenal glands were observed after MEX (Fig. 3); however, renalase mRNA contents in the kidney were significantly lower in the MEX group than that in the CON group (Fig. 3). In addition, renalase protein expression in the kidney decreased with MEX, which was consistent with the changes in the mRNA contents (Fig. 4). However, renalase mRNA contents in the EDL muscles were significantly higher in the MEX group than in the CON group, although there was no significant exercise‐induced difference in renalase mRNA contents between the plantaris and soleus muscles (Fig. 3). Moreover, renalase protein expression in the plantaris muscle increased after MEX (Fig. 4). However, there were no significant differences in renalase protein expression in the soleus or EDL muscles between the MEX and CON groups (Fig. 4). These results suggested that renalase expression in the skeletal muscle after MEX for 60 min differs depending on the muscle fiber type. Czarkowska‐Paczek *et al*. [13] showed that renalase mRNA contents in the white portion of the gastrocnemius muscle are higher than those in the red portion immediately after exercise at 28 m·min^−1^. Tokinoya *et al*. [11] reported that renalase expression in the soleus muscle increases after low‐intensity exercise, whereas in the white muscle‐dominant plantaris, renalase expression levels increase after high‐intensity exercise. Therefore, increases in renalase expression levels in skeletal muscles are related to exercise intensity and muscle fiber type [11]. In this study, renalase mRNA and protein expression levels were increased in various skeletal muscles at the same time point during MEX, because of the recruitment of both red and white muscle fibers.

The decreased expression of renalase in the kidney observed in this study is consistent with observations in previous studies [11, 12]. Blood flow in the kidney was limited because of the prioritization of flow to skeletal muscles during exercise. However, plasma renalase concentration increased significantly, presumably because of the increased expression of renalase in the skeletal muscles. In addition, renalase expression in each organ tested, except skeletal muscles, did not change after acute exercise.

We also examined the contribution of renalase expression to the metabolic function of catecholamine in skeletal muscles, because there is known to be an increase in the production of catecholamines during exercise [17]. In addition, it has been reported that overexpression of STAT3, Sp1 and ZBP89 increases renalase promoter activity. In other words, the absolute levels of these transcription factors are important determinants of renalase expression. Therefore, the mRNA contents of *STAT3*, *Sp1* and *ZBP‐89*, all of which are involved in the regulation of catecholamine, were also assessed to identify the CON mechanism for renalase expression during exercise. However, STAT3 is known to be activated when phosphorylated [19]. Thus, future research should also examine the potential mechanisms whereby STAT3 may activate the renalase promoter, because only *STAT3* mRNA content was measured in skeletal muscle in this study. Yoshida *et al*. [7] showed that renalase expression in C2C12 myotube cells is increased by epinephrine *in vitro*. Our results showed that the mRNA contents of these catecholamine regulators were significantly increased in the soleus muscle, whereas in the plantaris and EDL muscles, they did not change significantly (Fig. 5). Tokinoya *et al*. [11] suggested that renalase expression in the plantaris, in white or fast‐twitch fibers, is regulated by nuclear factor‐κB under conditions of oxidative stress. These results may differ from the effects of transcription factors on renalase expression observed in red and white fibers during exercise. Thus, catecholamine stimulation in the skeletal muscles, especially the soleus muscle, may influence renalase expression. However, the effects of these regulators on renalase promoter activation during exercise and the specific time points at which these occur are unclear. It is possible that these regulators are activated by acute exercise. In future studies, we will investigate the activation of these regulators and renalase promoter activity at various time points during acute exercise.

## Conclusions

The concentration of renalase in the blood and its expression in the skeletal muscle were increased after MEX for 60 min. However, renalase expression was not increased in the kidney or in the other tissues tested.

## Author contributions

K. Tokinoya and YY performed the experiments. K. Tokinoya and YY drafted the manuscript. K. Tokinoya, YY, TS and K. Takekoshi analyzed the data. All authors edited and revised the manuscript. All authors approved the final version of the manuscript.

## Conflict of interest

The authors declare no conflict of interest.
